# Nation-Wide Analysis of Glaucoma Medication Prescription in Fiscal Year of 2019 in Japan

**DOI:** 10.3390/jpm12060956

**Published:** 2022-06-11

**Authors:** Masaki Tanito

**Affiliations:** Department of Ophthalmology, Shimane University Faculty of Medicine, Izumo 693-8501, Shimane, Japan; mtanito@med.shimane-u.ac.jp; Tel.: +81-853-20-2284

**Keywords:** big data, gender difference, latanoprost, rho kinase inhibitor, omidenepag isopropyl

## Abstract

To report the updated prescription trend of antiglaucoma medications, the dose-based prescription of a glaucoma medication in Japan in the fiscal year 2019 was aggregated by using the National Database of Health Insurance Claims and Specific Health Checkups of Japan (NDB) Open data. Of the 100 most frequently prescribed topical medications for outpatients from out-hospital pharmacies, 32 glaucoma medications were identified. This year, 150.8 million ml of glaucoma medications prescribed accounted for 12.3% of the total prescription dose (1.3 billion ml). The dose was the largest with prostaglandin FP_2α_ agonist (PGF_2α_), followed by the fixed-dose combination (FDC) of β-blocker and carbonic anhydrase inhibitor (β + CAI) and α_2_-agonist. Prescription doses peaked at 75–79 years old for all medication classes, except for prostaglandin EP_2_ agonist of that peaked 10 years younger age class than other medications. The prescription dose was larger in women (55.3%) than men (44.7%), single medication formulation (71.2%) than FDC (28.8%), and brand-name (85.2%) than generic (14.8%). By multivariate analysis, prescription doses were affected by roles of the sex (*p* = 0.0066) and brand-name or generic (*p* = 0.032), but not by single medication formulation or FDC (*p* = 0.67); age was the most remarkable parameter for the difference in prescription dose (*p* < 0.0001). Dose-based anti-glaucoma medication prescription was analyzed using the government-provided most recent database on a national scale. The results provide the up-to-date real-world glaucoma medication prescriptions where the country has the highest aging rate in the world.

## 1. Introduction

Various previous studies have reported that, among the medications prescribed in the ophthalmology field, medications for the treatment of glaucoma or ocular hypertension account for the largest in both prescription dose and cost over medications for other eye diseases [1,2]. To date, analysis of glaucoma medication prescription was conducted by nationwide studies using the database from medical insurance claims of hospitals and pharmacies, or from a large-scale cohort study [3,4,5,6]. The trends of medication prescription have been changed by various factors such as the appearance of new medication classes and regimens [4,5,7,8], the appearance of alternative treatments other than medication [9,10,11], or an increase of the glaucoma patients and aged population [4,12,13]. Therefore, continuing updates for the database and its analysis are required to understand the current status of glaucoma medication usage.

The National Database of Health Insurance Claims and Specific Health Checkups of Japan (NDB), provided by the Ministry of Health and Welfare, contains all the insurance claims of the Japanese national healthcare system; representative data including nationwide medication prescriptions are public as NDB Open Data (https://www.mhlw.go.jp/stf/seisakunitsuite/bunya/0000177182.html) (accessed on 9 June 2022). In this manuscript, using the newest database of NDB Open Data (fiscal year 2019), analyzed results of the nationwide prescription of topical anti-glaucoma medication were reported. In the analyses, roles of patients’ age and sex, medication classes, single medication formulation or fixed-dose combination (FDC), and brand-name or generic were considered.

## 2. Materials and Methods

The study adhered to the tenets of the Declaration of Helsinki. Based on the Ethical Guidelines for Medical and Health Research Involving Human Subjects in Japan, given the study included open data sources only, ethical approval was not required. The 6th NDB Open Data, which contains the data from April 2019 to March 2020, was used. NDB Open Data provides the data on 100 most frequently prescribed ophthalmology medications, therefore this study did not contain the medications outside of the top 100 list. In each medication, the numbers of prescriptions were provided in each 5-year-step age of patients of each sex; the number of prescriptions fewer than 1000 in each age class was omitted and did not provide. In NDB Open Data, in- and out-hospital prescriptions for outpatients, and in-hospital prescriptions for hospitalized patients were separately provided; the dataset of out-hospital prescriptions for outpatients was analyzed in this study because the number of prescriptions was by far the largest for this database. In the top 100 list, 32 anti-glaucoma medications were identified. In the database, the data was provided by the ml volume for the bottled medications while by the number of medications for the unit dose medications. To enable direct comparisons among medications, for the unit dose medications, data were converted into prescription doses in ml by multiplying the number with volume of each unit (i.e., 0.3 or 0.4 mL per unit). The established dataset and list of 32 medications were available as Supplementary File (File S1).

The 32 medications were classified into 9 medication classes based on the mechanisms of IOP reduction (Table 1). To assess the effect of age, sex, single medication formulation or FDC, and brand-name or generic, mixed-effect regression model was used by these parameters were set as fixed effects, and 32 medications were set as variable effect. All the statistical analysis was performed using JPM Pro statistical software version 15.2 (SAS Institute, Inc., Cary, NC, USA).

## 3. Results

**Figure 1:**
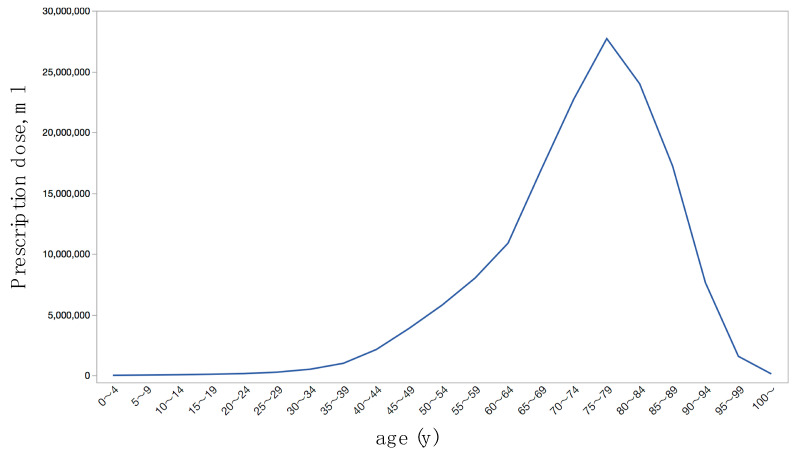
Age-dependent distributions of prescription dose of topical anti-glaucoma medications.

In the fiscal year of 2019, 150.8 million ml of anti-glaucoma medications were prescribed in the dataset of out-hospital prescriptions for outpatients in Japan. The prescription doses in each glaucoma medication class, and that stratified by sex, single medication formulation or FDC, and brand-name or generic were summarized in Table 2. The prescription dose was the largest with prostaglandin PGF_2α_ agonist (PGF_2α_), followed by FDC of β-blocker and carbonic anhydrase inhibitor (β + CAI) and α_2_-agonist. The prescription dose of each age class is shown in Figure 1; the data for the generation of figures in this manuscript was found in the Supplementary File (File S2). Among age classes, the prescription dose was the highest in the 75–79 years with 27.7 million mL.

**Figure 2:**
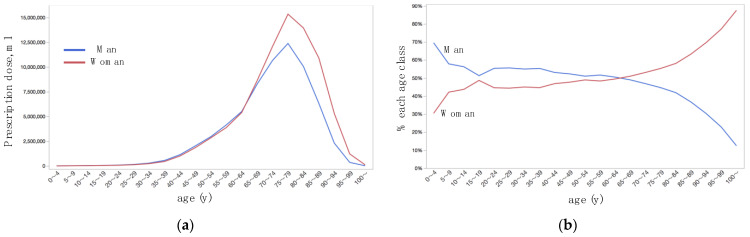
Age-dependent distributions of prescription dose of topical anti-glaucoma medications stratified by sex. (**a**) Actual prescription dose. (**b**) Percentage of prescription dose in each age class.

**Figure 3:**
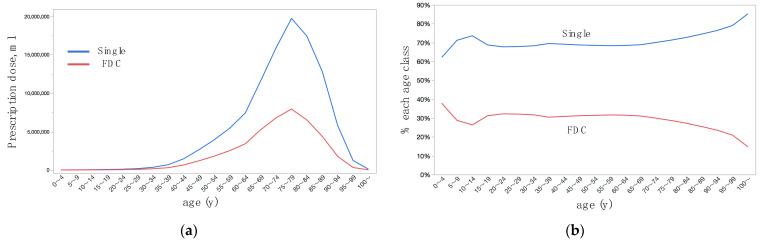
Age-dependent distributions of prescription dose of topical anti-glaucoma medications stratified by single medication formulation or fixed-dose combination. (**a**) Actual prescription dose. (**b**) Percentage of prescription dose in each age class. FDC, fixed-dose combination.

**Figure 4:**
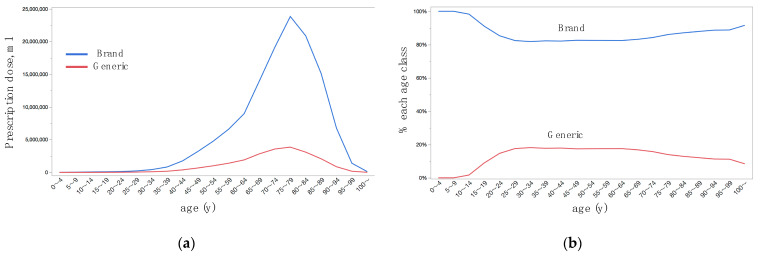
Age-dependent distributions of prescription dose of topical anti-glaucoma medications stratified by brand-name or generic. (**a**) Actual prescription dose. (**b**) Percentage of prescription dose in each age class.

Prescription doses were larger in women than men (Table 2, Figure 2a), single medication formulation medications than FDC (Table 2, Figure 3a), and brand-name than generic (Table 2, Figure 4a). By multivariate analysis (Table 3, Model 1), the difference in prescription doses were significant for sex (*p* = 0.0066) and brand-name or generic (*p* = 0.032), while were not significant for single medication formulation or FDC (*p* = 0.67); difference in age was the most remarkable parameter for the difference in prescription dose (*p* < 0.0001). Age-dependent changes in shares of prescription dose were shown for sex (Figure 2b), single medication formulation or FDC (Figure 3b), and brand-name or generic (Figure 4b). The prescription dose was larger in men for the age classes of 60–64 years or younger, while it was reversed at the age classes of 65–69 years or older (Figure 2b). Except for the very old age classes, the share was relatively stable for single medication formulation for FDC (Figure 3b). The share of generic was the highest at the age classes of 25–29 years and 30–34 years, became relatively stable after then, and gradually decreased at 70–74 years and older age classes (Figure 4b). By multivariate analysis (Table 3, Model 2), age-dependent changes in prescription dose were significant for sex (*p* = 0.0071) and brand-name or generic (*p* < 0.0001), while was not significant for single medication formulation or FDC (*p* = 0.91). Again, the difference in age class was the significant parameter for the difference in prescription dose (*p* < 0.0001).

**Figure 5:**
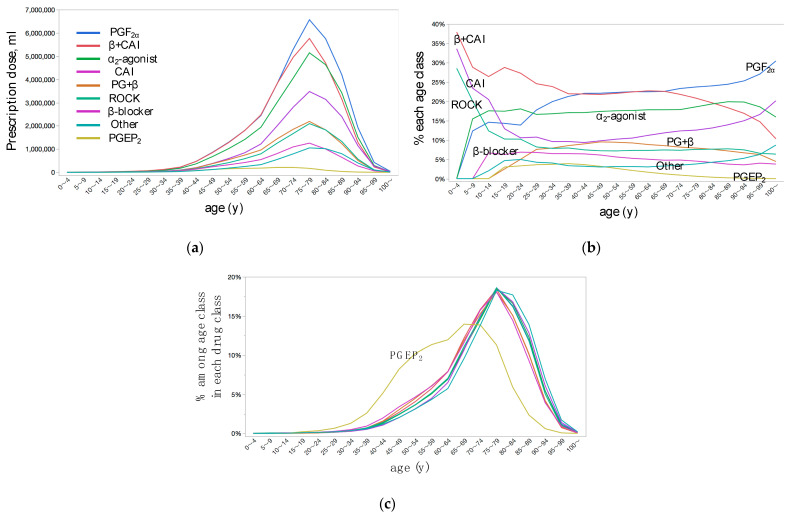
Age-dependent distributions of prescription dose of topical anti-glaucoma medications stratified by medication class. (**a**) Actual prescription dose. (**b**) Percentage of prescription dose in each age class. (**c**) Percentage of prescription dose in each age class in each medication class. PGF_2__α_, prostaglandin F_2α_ agonist; CAI, carbonic anhydrase inhibitor; β + CAI, foxed combination of β-blocker and CAI; PG + β, fixed combination of PGF_2__α_ and β-blocker; ROCK, rho kinase inhibitor; PGEP_2_, prostaglandin EP_2_ agonist.

Age-dependent prescription dose in each medication class and their share in each age class were shown in Figure 5a,b, respectively. For very young age classes, β ± CAI, CAI, and ROCK were the major choice of medications, while PGF_2α_, α_2_-agonist, and CAI were the major choice for very old age classes (Figure 5b). In each class of medication, the prescription dose peaked at the 75–79 years age class, while the peak was seen at 65–69 years old age class for PGEP_2_ agonist (PGEP_2_) (Figure 5c).

## 4. Discussion

By analyzing the governmental medication prescription database, at least 150.8 million mL of anti-glaucoma medications were prescribed in the fiscal year of 2019 in Japan. This accounts for 12.3% of the total prescription dose (1.23 billion ml) of the top 100 medication list for an out-hospital prescription for outpatients. In the USA, through Medicare Part D in 2013, the total cost driven by glaucoma medications, accounted for 54% of total medication cost and 72% of total volume [1]. In a retrospective, longitudinal cohort study from 2015 through 2016 in the USA, glaucoma medications accounted for 42.7% of all ophthalmic medication expenditures, that followed by dry eye medications (29.5%) [2]. In Japan from the current study, among the 100 most frequently prescribed ophthalmology medications, 32 glaucoma medications were included (Table 1). In England, by prescribing cost analysis data held by NHS Business Authority from 2000 to 2012, the number of glaucoma medication prescriptions dispensed increased by 67% in 2012 from 2000. Over the same time period, medication costs increased by 88% [3]. In Australia, the number of glaucoma medication prescriptions peaked in 2015, but then declined by 14.9% in 2017, this was accompanied by the substantial increment of glaucoma laser therapies, drainage device implantations, and trabecular microbypass surgeries over the same period [6]. Although this study did not provide the trend, the current study must be close to a real-life picture of the most recent use of glaucoma medications on a national scale.

Among 5-year step age classes, the prescription dose was the highest in the 75–79 years (Figure 1), and the amounts were larger in women than men (Table 2, Figure 2a). Age, sex, and interaction between age and sex were associated with the prescription dose (Table 3). In the USA, of 3826 patients with glaucoma selected from the nationwide longitudinal cohort, the median age group was 60–75 years, and 60.3% were women [14]. In the USA from 2004 to 2009, the proportion of the population with ocular hypotensive treatment was 11.5% while was 1.2% in 50–64 year, thus the proportion suddenly increased in the 65–79 years group and older [13]. In Glaucoma/OHT prescription statistics for England and its constituent primary care trusts, the change in prescription rates between 2008 and 2012 was correlated with age [15]. Patients who were older than 65 years were significantly more likely to have greater out-of-pocket spending on ophthalmic medications [2]. Accordingly, our results were explained by the age-dependent increase in glaucoma prevalence [4,12] and by the population composition of Japan (more older women than men). Patients who placed the eye drops outside the eye required more numbers of per month prescription bottles than the patients with good eyedrop usage skills [16]. The mean age was significantly higher in the eyedrop-usage failure group than in the success group for the sitting position [17]. Thus, more requirements of bottles per patient might be involved in the rapid increase of prescription dose in the 60–64 years group and older in our dataset. It is interesting to note that, the prescription dose was larger in men for the age classes of 60–64 years or younger, while it was reversed at the age classes of 65–69 years or older (Figure 2b). This might be explained by the difference in actual glaucoma prevalence, but the other socio-economic reasons such as the gender differences in the chance of early detection of glaucoma through the company- or employer-provided health check-up programs, since the employment rate was higher in men than women in Japan. 

The prescription dose was the largest with PGF_2α_, followed by β + CAI FDC and α_2_-agonist (Table 2); among the PGF_2α_, brand-name and generic latanoprost were the most common (File S1). After marketing the PGF_2α_, the curves of market shares between PGF_2α_ and β-blocker crossed in 2002 in Taiwan [5], by 2004 in Scotland [7], and were predicted to be crossed in 2005 in five European countries [8]. In Denmark from 1996 to 2011, the use of PGF_2α_ and FDCs increased, whereas the use of β-blockers, CAIs and parasympathomimetic medications decreased; the use of α_2_-agonists remained unchanged [4]. In England during 2000–2012, prescriptions for PGF_2α_ increased 4-fold, while there was a 3-fold decrease in the use of β-blockers; the most commonly prescribed glaucoma medication was latanoprost [3]. In six major cities of China, β-blockers were the most commonly prescribed in 2013, accounting for 34.3% of patients, but gradually decreased to 27.1% in 2017; PGF_2α_ became the most frequently prescribed medication in 2017, accounting for 30.2% of the visits [18]. In Japan where the first PGF_2α_ latanoprost appeared in the market in 1999, the situation seemed on the same trend as the countries worldwide. In the Japan Health Insurance Society database who were newly diagnosed with glaucoma, initial prescription of PGF_2α_ showed significantly better patient persistence than β-blockers and CAIs [19]. In Australia, decrease of single-agent β-blockers was particular with the introduction of FDCs, which noted an upward trend [10]. Among patients who were prescribed PGF_2α_ as an initial medical treatment, 30% of the patients required adjunctive therapy within 1 year, and FDCs were the most common initial adjunctive therapy [20]. By using electronic dose monitors in glaucoma patients, younger age and higher bottle numbers of eye diseases were associated with poor treatment adherence [21]. The interaction between usage of single or FDC and age was not associated with prescription dose (Table 3). Accordingly, good efficacy and persistence/adherence of PGF_2α_ and FDCs likely explained the common use of these medication classes irrespective of age classes.

The use of generic medicines has grown considerably in recent years providing considerable cost savings. In our dataset, the share of generic medication dose was 14.8% in total (Table 2). In India in 2011, medications were predominantly prescribed in brand names 83% instead of generic names [22]. In USA, brand/generic cost ratio was 75%/25% for glaucoma medication in 2013 [1]. In England, generic medications represented 11.7% of prescriptions for glaucoma and ocular hypertension in 2009, increasing to 55.2% of prescriptions in 2018 [23,24]; the introduction of generic latanoprost in 2012 more than halved the cost associated with this medication in England [3]. Accordingly, the results indicate the predominance of brand-name medications over generic medications in Japan. Bioequivalence between generic and innovator compounds is presumed on the basis of matching active and inactive ingredient profiles, therefore the generic compounds may differ from innovator agents with regards to inactive ingredients, pH, viscosity, levels of particulate matter, degradation over time, bottle design, and rigidity [23,25]. The national mentality of Japanese on reliability preference rather than cost-benefit might associated with the low share of generic medication use even in 2019. Use of the brand name or generic was affected by age (Table 3, model 2); the use of brand-name was relatively high in children and older age classes, while the use of generic medications was high in working generations (Figure 4b). In USA, female sex, younger age, and lower-income (<$30,000) were associated with the cost-related nonadherence to glaucoma medical treatment [13]. In Japan under the public insurance, the burden rate for prescribed medications was lower in children before schooling and after the age of 70 years. Thus, the difference in out-of-pocket cost likely explains the age-dependent difference in prescription dose in this study.

In the youngest age glass, β + CAI, CAI, and ROCK were the major choices of drugs, while PGF_2α_ and α_2_-agonist were merely used (Figure 5b). In children, the IOP-lowering effects were equivalent among glaucoma medicine classes, while the β ± CAI had the greatest persistence [26]. Because of the prematurity of blood-brain barrier, brimonidine tartrate can cause drowsiness due to central nervous system depression [26]. In Japan, brimonidine tartrate was contraindicated in children under the age of 2 years, and not recommended in children under the age of 6 years. Prescription of glaucoma medication to the outpatients from the out-of-the-hospital pharmacies itself is not common for the youngest age class. The NDB Open Data did not provide the data when the prescription dose was less than 1000 in each age class, accordingly, this might explain the zero prescription of PGF_2α_ in this age class. Very old age classes, PGF_2α_, α_2_-agonist, and CAI were the major choice, while β-+CAI and β-blocker reduced share (Figure 5b). This was explained by the care for weakened tolerance due to decrement of cardiovascular/pulmonary functions in this age class. For all medication classes, the prescription dose peaked at 75–79 years while the peak was seen at 65–69 years for PGEP_2_ (Figure 5c). The first PGEP_2_ omidenepag isopropyl (OMDI) was introduced in November 2018 in Japan. OMDI 0.002% was non-inferior to latanoprost 0.005% in reducing IOP in glaucoma patients [27], therefore has been used as a first-line medication. Because of the possible risk of macular edema, OMDI is contraindicated in patients with pseudophakic eyes [28]. This is a possible explanation of the unique distribution of PGEP_2_. OMDI reduces IOP by enhancing both conventional trabecular and uveoscleral outflow facilities without prostaglandin-associated periorbitopathy (PAP) seen with PGF_2α_ [28,29]. By switching from PGF_2α_ to OMDI, signs of PAP were improved within 3 months after the switching [30,31]. Accordingly, preferable use by relatively young generations who care about the cosmetic side effect likely explained the distribution of PGEP_2_. 

As described previously, the database analyzed did not include the medications outside of the top 100 prescription list, the prescription fewer than 1000 in each medication of 5-year-step age of each sex, and in-hospital prescriptions. In the fiscal year 2019, the total dose of eyedrop prescription (excluding ointments, viscoelastic devices, and surgical irrigation solutions) were 1.23 billion ml for an out-hospital prescription for outpatients, 415 million ml for in-hospital prescriptions for outpatients, and 30.0 million ml for an in-hospital prescription for hospitalized patients (https://www.mhlw.go.jp/stf/seisakunitsuite/bunya/0000177182.html) (accessed on 9 June 2022). Given the current database contains the majority of the total prescriptions, I can imagine that the results might not change largely if the missing data indicated above were included in the analyses. In general, the medication price per unit is much more expensive with glaucoma medications than other topical medications, thus the share of glaucoma medications over other medications should increase if the aggregation was performed by cost. In the same context, the shares of recently marketed medications and brand-name can increase in the cost-based analyses. 

## 5. Conclusions

Dose-based anti-glaucoma medication prescription was analyzed using the government-provided most recent database. Glaucoma medication prescription was affected by the roles of age, sex, and use of generic medications. The results provide the real-world glaucoma medication prescriptions where the country has the highest aging rate in the world (https://www.stat.go.jp/data/topics/topi1261.html) (accessed on 9 June 2022).

## Figures and Tables

**Table 1:** Demographics of topical anti-glaucoma medications included in this study.

Class	Total	Single	FDC	Brand	Generic
PGF_2α_	10	10	-	5	5
β + CAI	6	-	6	3	3
α_2_-agonist	1	1	-	1	-
CAI	4	4	-	2	2
PG + β	4	-	4	4	-
ROCK	1	1	-	1	-
β-blocker	3	3	-	2	1
PGEP_2_	1	1	-	1	-
Other	2	2	-	2	-
Total	32	22	10	21	11

Single. single medication formulation; FDC, fixed-dose combination; Brand, brand-name medication; PGF_2α_, prostaglandin FP_2α_ agonist; CAI, carbonic anhydrase inhibitor; β + CAI, foxed combination of β-blocker and CAI; PG + β, fixed combination of PGF2α and β-blocker; ROCK, rho kinase inhibitor; PGEP_2_, prostaglandin EP_2_ agonist.

**Table 2:** Total prescription dose and prescription dose of each medication class.

Class	Total	Man	Woman	Single	FDC	Brand	Generic
PGF_2α_	35,310,214	15,327,512	19,982,703	35,310,214	-	26,975,993	8,334,221
β + CAI	31,298,874	14,252,703	17,046,171	-	31,298,874	24,087,264	7,211,610
α_2_-agonist	27,871,841	13,159,152	14,712,689	27,871,841	-	27,871,841	-
CAI	18,712,262	8,344,612	10,367,650	18,712,262		13,887,168	4,825,094
PG + β	12,119,545	5,518,501	6,601,044	-	12,119,545	12,119,545	-
ROCK	11,273,786	5,580,787	5,692,999	11,273,786	-	11,273,786	-
β-blocker	6,947,340	2,557,882	4,389,458	6,947,340	-	5,052,487	1,894,853
PGEP_2_	1,500,846	529,063	971,783	1,500,846	-	1,500,846	-
Other	5,748,294	2,060,808	3,687,486	5,748,294	-	5,748,294	-
Total	150,783,002	67,331,020	83,451,983	107,364,583	43,418,419	128,517,224	22,265,778

Data are expressed in ml. Single. single medication formulation; FDC, fixed-dose combination; Brand, brand-name medication; PGF_2__α_, prostaglandin F_2α_ agonist; CAI, carbonic anhydrase inhibitor; β + CAI, FDC of β-blocker and CAI; PG + β, FDC of PGF_2__α_ and β-blocker; ROCK, rho kinase inhibitor; PGEP_2_, prostaglandin EP2 agonist.

**Table 3:** Multivariate analysis by mixed-effect regression models.

Variables	F Value	*p*-Value
**Model 1**		
Age class	49.6	<0.0001
Sex	7.4	0.0066
Single or FDC	0.2	0.67
Brand or Generic	5.1	0.032
**Model 2**		
Age class	30.2	<0.0001
Sex * Age class	2.0	0.0071
Single or FDC * Age class	0.6	0.91
Brand or Generic * Age class	10.9	<0.0001

Single. single medication formulation; FDC, fixed-dose combination; Brand, brand-name medication; * interaction between two variables.

## Data Availability

All the relevant data used in this study were included in this manuscript or available as Supplementary Files S1 and S2.

## Supplementary Materials

The following supporting information can be downloaded at: https://www.mdpi.com/article/10.3390/jpm12060956/s1, File S1: Full dataset underlying this manuscript; File S2: Data used for generating figures.
