# Supplementary material for: Nation-Wide Analysis of Glaucoma Medication Prescription in Fiscal Year of 2019 in Japan
Source: J Pers Med. 2022 Jun 11;12(6):956. doi: 10.3390/jpm12060956 (PMC9224924; doi:10.3390/jpm12060956)
Supplement: Supplementary file 1 [file jpm-12-00956-s001.zip › Supplementary files/File S1.pdf]

CODE	Drug name JPN	Drug name ENG	Class	FDC
622,150,001	アイファガン点眼液０．１％	AIPHAGAN OPHTHALMIC SOLUTION 0.1%	α2	-
621,984,801	コソプト配合点眼液	COSOPT ophthalmic solution	β+CAI	1
622,374,601	グラナテック点眼液０．４％	GLANATEC ophthalmic solution 0.4%	ROCK	-
622,418,901	コソプトミニ配合点眼液　０．４ｍＬ	COSOPT Mini ophthalmic solution	β+CAI	1
620,224,801	キサラタン点眼液０．００５％	Xalatan Eye Drops 0.005%	PG	-
620,008,568	タプロス点眼液０．００１５％	TAPROS ophthalmic solution	PG	-
620,005,257	エイゾプト懸濁性点眼液１％	Azopt Ophthalmic Suspension 1%	CAI	-
622,278,801	アゾルガ配合懸濁性点眼液	AZORGA Combination Ophthalmic Suspension	β+CAI	1
622,248,201	タプロスミニ点眼液０．００１５％　０．３ｍＬ	TAPROS Mini ophthalmic solution	PG	-
660,432,003	トルソプト点眼液１％	TRUSOPT ophthalmic solution 1%	CAI	-
622,509,901	ミケルナ配合点眼液	Mikeluna combination ophthalmic solution	PG+β	1
621,933,101	ルミガン点眼液０．０３％	LUMIGAN OPHTHALMIC SOLUTION 0.03%	PG	-
620,004,892	ミケランＬＡ点眼液２％	Mikelan LA ophthalmic solution 2%	β	-
620,005,833	トラバタンズ点眼液０．００４％	TRAVATANZ Ophthalmic Solution 0.004%	PG	-
622,640,201	ドルモロール配合点眼液「ニットー」	Dormolol Combination Ophthalmic Solution 「NITTO」	β+CAI	1
620,005,312	レスキュラ点眼液０．１２％	Rescula Eye Drops 0.12%	Other	-
660,451,004	デタントール０．０１％点眼液	Detantol ophthalmic solution	Other	-
622,279,101	タプコム配合点眼液	TAPCOM combination ophthalmic solution	PG+β	1
622,640,101	ブリンゾラミド懸濁性点眼液１％「ニットー」	Brinzolamide Ophthalmic Suspension 1%「NITTO」	CAI	-
622,637,201	ドルモロール配合点眼液「センジュ」	DORMOLOL COMBINATION OPHTHALMIC SOLUTION「SENJYU」	β+CAI	1
621,974,201	ザラカム配合点眼液	Xalacom Combination Eye Drops	PG+β	1
621,985,001	デュオトラバ配合点眼液	DUOTRAV Combination Ophthalmic Solution	PG+β	1
622,637,101	ブリンゾラミド懸濁性点眼液１％「センジュ」	BRINZOLAMIDE OPHTHALMIC SUSPENSION 1%「SENJU」	CAI	-
621,990,401	ラタノプロスト点眼液０．００５％「ニットー」	Latanoprost Ophthalmic Solution 0.005%「NITTO」	PG	-
622,282,101	カルテオロール塩酸塩ＬＡ点眼液２％「わかもと」	Carteolol Hydrochloride LA Ophthalmic Solution 2%「WAKAMOTO」	β	-
621,980,502	ラタノプロスト点眼液０．００５％「ＳＥＣ」	Latanoprost ophthalmic solution 「SEC」	PG	-
620,003,860	チモプトール点眼液０．５％	TIMOPTOL ophthalmic solution 0.5%	β	-
622,640,001	トラボプロスト点眼液０．００４％「ニットー」	Travoprost Ophthalmic Solution 0.004%「NITTO」	PG	-
621,984,601	ラタノプロスト点眼液０．００５％「センジュ」	LATANOPROST OPHTHALMIC SOLUTION 0.005% 「SENJYU」	PG	-
622,621,701	ドルモロール配合点眼液「わかもと」	LATANOPROST OPHTHALMIC SOLUTION 0.005%「WAKAMOTO」	β+CAI	1
622,653,101	エイベリス点眼液０．００２％	EYBELIS ophthalmic solution	EP2	-
621,996,301	ラタノプロストＰＦ点眼液０．００５％「日点」	Latanoprost PF Ophthalmic Solution 0.005%「NITTEN」	PG	-

Generic	Man													
	0-4	5-9	10-14	15-19	20-24	25-29	30-34	35-39	40-44	45-49	50-54	55-59	60-64	65-69
-	0	3695	6030	9280	15900	25140	48150	96250	198975	368684	550607	781160	1050178	1576045
-	2145	3700	6170	7150	8600	13615	23650	43150	82345	148370	211789	300135	399149	629538
-	1980	4255	3700	5888	9010	13980	25195	49300	92805	162675	241495	338631	445380	676249
-	0	0	448	1426	1890	4170	4812	11473	20613	43317	57323	87023	115414	183194
-	0	2748	3863	3358	4015	6735	13908	28275	57418	99890	148955	209008	275045	419513
-	0	0	1613	2870	3813	6613	14193	30803	69635	118014	170063	238298	308053	458490
-	1220	3680	4305	3330	4020	7230	11420	21595	43185	85173	125500	177173	244890	404184
-	1140	1760	2500	3625	5640	9110	17900	37060	75590	133355	196898	270025	363830	540907
-	0	0	420	524	614	1228	2576	5348	10501	17028	23060	36080	49288	77869
-	1415	2070	2900	2270	2300	3920	6925	13635	24535	48600	72995	104675	154745	256563
-	0	0	0	1198	2595	5690	10960	21668	49130	84058	119168	162573	201226	284240
-	0	0	0	1293	2515	3427	6720	12513	24110	48558	73258	104868	142630	219343
-	0	0	1410	1948	2540	3533	6705	12605	27344	48014	63970	83969	101685	141283
-	0	0	0	0	1120	2188	3733	8908	19918	35480	51958	73898	100171	156315
1	0	0	0	1775	3295	5215	9693	18125	36343	62420	88218	124078	166455	227068
-	0	0	1335	2190	3790	4530	7910	11105	20755	33135	44893	58842	74190	120655
-	0	0	0	0	0	1530	2990	4575	10650	18130	32920	47300	66127	106621
-	0	0	0	0	1038	2283	4973	11268	24190	45312	65850	90955	111640	156275
1	0	0	0	1660	1645	2910	5255	10105	19405	37125	57785	78010	112425	168868
1	0	0	0	1205	1980	3420	6220	9835	23140	42010	58063	82556	119458	180898
-	0	0	0	0	1090	2095	4130	9150	16985	36055	51178	73205	93400	138974
-	0	0	0	0	1275	1770	3678	7910	17610	34258	51790	76902	93897	136382
1	0	0	1120	0	1250	1995	4145	7210	14865	27950	47428	72420	98800	150070
1	0	0	0	0	1250	2235	4663	9178	18265	30165	43035	56363	77475	111193
1	0	0	0	0	1786	3008	5785	11518	22198	35948	46698	61166	77638	101433
1	0	0	0	0	0	1683	3665	7473	16885	27538	41485	57861	76155	108543
-	0	0	1213	1210	0	1905	2910	5590	11033	19013	25578	35170	49810	78060
1	0	0	0	0	0	1470	3278	6453	14595	24565	38193	52910	69940	97895
1	0	0	0	0	0	1400	2740	5938	13883	25279	34035	49183	64055	97328
1	0	0	0	0	1680	2280	4880	7520	16380	26890	43808	59140	78488	115310
-	0	0	0	1428	2150	4503	8198	15508	30709	44965	53909	61342	64368	71184
1	0	0	0	0	0	1638	3058	5823	12721	20415	28031	37830	52241	75130

								Woman						
70-74	75-79	80-84	85-89	90-94	95-99	100-	Total	0-4	5-9	10-14	15-19	20-24	25-29	30-34
2043438	2461637	2038906	1322587	487808	71307	3375	13159152	0	2155	5515	8935	12365	20405	38830
857364	1025981	840196	528954	195148	26876	1525	5355550	1020	2980	4663	7015	7870	10810	18708
863506	1031723	849532	538858	196855	28050	1720	5580787	1260	3340	4425	4810	6990	8465	15339
197831	244898	201840	106672	34275	3557	0	1320177	0	839	764	1442	995	3699	7294
587710	723972	608277	392622	149481	26800	1705	3763298	0	1923	2578	3415	3575	5310	10618
611962	717400	582770	356847	131560	20267	1855	3845119	0	0	0	1663	2739	5224	10765
586478	727528	636827	433799	166495	31190	2155	3721377	1180	3105	3540	3175	3135	4645	8235
650190	702926	518637	296350	99955	13910	0	3941308	0	1625	2830	3970	4880	7345	15740
94869	124275	101274	63555	23471	3309	359	635648	0	0	0	615	498	1191	2485
351168	442620	396440	271775	109335	20220	1405	2290511	0	0	1605	2030	2130	3465	6160
331723	355030	264763	146910	45984	5520	0	2092436	0	0	0	1410	1820	4168	9133
296998	368610	312056	211835	85598	14470	1000	1929802	0	0	1120	1195	1563	2683	4813
186160	208546	157292	87845	29955	5303	0	1170107	0	0	1753	2073	2883	4216	9330
225208	268973	235438	155738	60038	9343	0	1408427	0	0	0	0	0	1300	3440
265525	271720	195821	116170	35554	4480	0	1631955	0	0	0	1425	3180	3755	7053
159380	192584	176320	113253	48575	7600	1055	1082097	0	0	0	1655	2720	3985	6960
149865	186927	168403	123308	48820	10545	0	978711	0	0	0	1030	1260	1560	3070
189428	204110	155468	90230	28180	3675	0	1184875	0	0	0	0	0	1863	3368
207222	215245	164755	103228	34145	5855	0	1225643	0	0	0	1015	1050	2483	4365
211315	204798	153305	86550	27890	3460	0	1216103	0	0	0	1010	1405	2135	4473
179552	211350	166749	105637	40501	6025	0	1136076	0	0	0	0	0	1228	2680
175522	194831	160756	103875	37778	6880	0	1105114	0	0	0	0	0	1655	2025
190680	199145	155033	96645	33270	5055	0	1107081	0	0	0	0	1005	2795	3140
143538	156463	116373	71885	25280	4513	0	871874	0	0	0	0	0	1570	2860
120148	115645	76758	39785	11288	1850	0	732652	0	0	0	1288	2223	3683	6168
134410	144438	111638	64700	22623	2695	0	821792	0	0	0	0	0	1433	2590
104215	121045	105451	63170	24905	4845	0	655123	0	0	0	0	1295	2218	2641
126288	133804	103820	62118	20198	4060	0	759587	0	0	0	0	0	0	1960
119832	129960	96660	56513	19435	3568	0	719809	0	0	0	0	0	0	2378
134715	131608	89963	53973	18710	2265	0	787610	0	0	0	0	1310	1748	3010
72094	55800	29120	11285	2500	0	0	529063	0	0	0	1703	3020	5374	11003
91348	101008	79068	45570	15915	2360	0	572156	0	0	0	0	0	1343	2838

35-39	40-44	45-49	50-54	55-59	60-64	65-69	70-74	75-79	80-84	85-89	90-94	95-99	100-
75460	166750	306684	466921	632149	889147	1433087	2016969	2682713	2593019	2101952	1021348	221100	17185
30070	65014	119715	181372	265233	374699	634910	958494	1256105	1127541	879535	417767	88665	6455
30465	66565	117250	173918	246993	350338	575185	806802	1063505	975451	789590	370643	75195	6470
9785	26435	50957	99390	136241	198546	288976	381756	466640	368750	242911	94436	14771	980
21691	51967	93543	146616	209304	290154	486808	746141	1002816	935191	761216	395625	102552	11560
24710	55330	106505	165303	224303	307032	496175	708758	920870	850497	666205	322810	78180	7732
18665	40585	79740	128225	175841	269253	458426	681787	927461	883648	738323	389687	99170	12271
28695	67575	124915	188335	255085	359913	557420	731600	849523	690988	480891	203307	39170	2175
5902	14035	27136	49919	66257	89263	141845	198335	259282	219740	161433	72222	12017	895
10503	25160	41325	64203	96486	153020	268325	405713	526311	525746	469023	266528	69920	7530
18178	42898	80560	120643	155744	207197	317564	414433	501831	413608	278979	110375	20800	2045
7578	18433	35643	53645	74273	106065	170030	255715	341580	337705	298271	162203	37580	3450
16663	37259	69066	97505	121260	158506	244880	347343	425135	347693	232579	100670	24663	2243
6873	15015	32458	50396	72635	104265	172892	264797	356701	347974	288437	157267	40568	4663
12170	26815	41918	70663	95955	127753	192500	247875	260218	223798	158116	71915	15215	1120
11425	24165	41509	59470	81605	104140	178710	264370	363427	361464	289228	148057	40825	5370
6490	12625	24385	43160	61765	85790	145005	220731	306960	310640	273340	156125	39685	4780
7790	18356	38370	57653	75060	99583	156935	207258	253492	220181	154857	64413	13750	0
8690	17795	34558	51105	72795	98670	154550	199885	221030	195990	145698	77123	15810	1220
7725	17645	30155	49064	73975	106398	166615	199063	214529	188408	128165	54505	13275	1045
5388	12380	25730	40150	52105	77548	130018	186550	250082	231688	178486	90248	20835	1645
4650	10980	24663	38962	53544	75847	120325	168381	217909	213177	173882	91221	20719	2028
6185	14415	25870	44445	63660	87335	135575	181813	218277	182565	141353	64030	14645	1430
6248	14648	27595	41183	54240	75286	117750	162415	189429	163628	132590	64365	16410	2328
12971	27560	46213	63880	78981	101893	148240	191515	198465	145670	89526	36395	7530	0
5585	12125	23665	35455	46298	70533	107415	147598	173570	152498	114644	50308	12409	0
5725	11630	18968	26715	40240	57258	92028	144563	186385	168734	132654	69053	18805	2625
4405	10335	20115	31925	43525	58920	94545	119505	144493	127803	102690	50314	11428	1138
4393	10608	21133	29688	43825	61193	95421	128373	143015	131026	95880	49043	12723	1348
5235	10506	22313	33300	48060	61625	95015	120075	132845	104453	76473	32880	6060	0
23526	46873	78447	99636	108720	114966	138150	135652	113625	59562	23873	6623	1030	0
5885	12010	23500	35938	51370	66375	103558	136038	165770	140058	102406	47605	11225	1265

	Man+Woman											
Total	0-4	5-9	10-14	15-19	20-24	25-29	30-34	35-39	40-44	45-49	50-54	55-59	60-64
14712689	-	5,850	11,545	18,215	28,265	45,545	86,980	171,710	365,725	675,368	1,017,528	1,413,309	1,939,325
6458641	3,165	6,680	10,833	14,165	16,470	24,425	42,358	73,220	147,359	268,085	393,161	565,368	773,848
5692999	3,240	7,595	8,125	10,698	16,000	22,445	40,534	79,765	159,370	279,925	415,413	585,624	795,718
2395606	-	839	1,212	2,868	2,885	7,869	12,106	21,258	47,048	94,274	156,712	223,264	313,960
5282603	-	4,671	6,441	6,773	7,590	12,045	24,526	49,966	109,385	193,433	295,571	418,312	565,199
4954801	-	-	1,613	4,533	6,552	11,837	24,958	55,513	124,965	224,519	335,366	462,601	615,085
4930097	2,400	6,785	7,845	6,505	7,155	11,875	19,655	40,260	83,770	164,913	253,725	353,014	514,143
4615982	1,140	3,385	5,330	7,595	10,520	16,455	33,640	65,755	143,165	258,270	385,233	525,110	723,743
1323070	-	-	420	1,139	1,112	2,420	5,061	11,250	24,536	44,164	72,980	102,337	138,551
2945183	1,415	2,070	4,505	4,300	4,430	7,385	13,085	24,138	49,695	89,925	137,198	201,161	307,765
2701386	-	-	-	2,608	4,415	9,858	20,093	39,846	92,028	164,618	239,811	318,317	408,423
1913545	-	-	1,120	2,488	4,078	6,110	11,533	20,091	42,543	84,201	126,903	179,141	248,695
2245720	-	-	3,163	4,021	5,423	7,749	16,035	29,268	64,603	117,080	161,475	205,229	260,191
1919681	-	-	-	-	1,120	3,488	7,173	15,781	34,933	67,938	102,354	146,533	204,436
1561444	-	-	-	3,200	6,475	8,970	16,746	30,295	63,158	104,338	158,881	220,033	294,208
1989085	-	-	1,335	3,845	6,510	8,515	14,870	22,530	44,920	74,644	104,363	140,447	178,330
1698401	-	-	-	1,030	1,260	3,090	6,060	11,065	23,275	42,515	76,080	109,065	151,917
1372929	-	-	-	-	1,038	4,146	8,341	19,058	42,546	83,682	123,503	166,015	211,223
1303832	-	-	-	2,675	2,695	5,393	9,620	18,795	37,200	71,683	108,890	150,805	211,095
1259590	-	-	-	2,215	3,385	5,555	10,693	17,560	40,785	72,165	107,127	156,531	225,856
1306761	-	-	-	-	1,090	3,323	6,810	14,538	29,365	61,785	91,328	125,310	170,948
1219968	-	-	-	-	1,275	3,425	5,703	12,560	28,590	58,921	90,752	130,446	169,744
1188538	-	-	1,120	-	2,255	4,790	7,285	13,395	29,280	53,820	91,873	136,080	186,135
1072545	-	-	-	-	1,250	3,805	7,523	15,426	32,913	57,760	84,218	110,603	152,761
1162201	-	-	-	1,288	4,009	6,691	11,953	24,489	49,758	82,161	110,578	140,147	179,531
956126	-	-	-	-	-	3,116	6,255	13,058	29,010	51,203	76,940	104,159	146,688
981537	-	-	1,213	1,210	1,295	4,123	5,551	11,315	22,663	37,981	52,293	75,410	107,068
823101	-	-	-	-	-	1,470	5,238	10,858	24,930	44,680	70,118	96,435	128,860
830047	-	-	-	-	-	1,400	5,118	10,331	24,491	46,412	63,723	93,008	125,248
754908	-	-	-	-	2,990	4,028	7,890	12,755	26,886	49,203	77,108	107,200	140,113
971783	-	-	-	3,131	5,170	9,877	19,201	39,034	77,582	123,412	153,545	170,062	179,334
907184	-	-	-	-	-	2,981	5,896	11,708	24,731	43,915	63,969	89,200	118,616

65-69	70-74	75-79	80-84	85-89	90-94	95-99	100-	Total
3,009,132	4,060,407	5,144,350	4,631,925	3,424,539	1,509,156	292,407	20,560	27,871,841
1,264,448	1,815,858	2,282,086	1,967,737	1,408,489	612,915	115,541	7,980	11,814,191
1,251,434	1,670,308	2,095,228	1,824,983	1,328,448	567,498	103,245	8,190	11,273,786
472,171	579,588	711,538	570,590	349,582	128,712	18,328	980	3,715,783
906,321	1,333,851	1,726,788	1,543,468	1,153,838	545,106	129,352	13,265	9,045,901
954,665	1,320,720	1,638,270	1,433,267	1,023,052	454,370	98,447	9,587	8,799,920
862,610	1,268,265	1,654,989	1,520,475	1,172,122	556,182	130,360	14,426	8,651,474
1,098,327	1,381,790	1,552,449	1,209,625	777,241	303,262	53,080	2,175	8,557,290
219,714	293,203	383,557	321,013	224,988	95,694	15,327	1,254	1,958,717
524,888	756,881	968,931	922,186	740,798	375,863	90,140	8,935	5,235,694
601,804	746,156	856,861	678,371	425,889	156,359	26,320	2,045	4,793,822
389,373	552,713	710,190	649,761	510,106	247,801	52,050	4,450	3,843,347
386,163	533,503	633,681	504,985	320,424	130,625	29,966	2,243	3,415,827
329,207	490,005	625,674	583,412	444,175	217,305	49,911	4,663	3,328,108
419,568	513,400	531,938	419,619	274,286	107,469	19,695	1,120	3,193,399
299,365	423,750	556,011	537,784	402,481	196,632	48,425	6,425	3,071,182
251,626	370,596	493,887	479,043	396,648	204,945	50,230	4,780	2,677,112
313,210	396,686	457,602	375,649	245,087	92,593	17,425	-	2,557,804
323,418	407,107	436,275	360,745	248,926	111,268	21,665	1,220	2,529,475
347,513	410,378	419,327	341,713	214,715	82,395	16,735	1,045	2,475,693
268,992	366,102	461,432	398,437	284,123	130,749	26,860	1,645	2,442,837
256,707	343,903	412,740	373,933	277,757	128,999	27,599	2,028	2,325,082
285,645	372,493	417,422	337,598	237,998	97,300	19,700	1,430	2,295,619
228,943	305,953	345,892	280,001	204,475	89,645	20,923	2,328	194,419
249,673	311,663	314,110	222,428	129,311	47,683	9,380	-	1,894,853
215,958	282,008	318,008	264,136	179,344	72,931	15,104	-	1,777,918
170,088	248,778	307,430	274,185	195,824	93,958	23,650	2,625	1,636,660
192,440	245,793	278,297	231,623	164,808	70,512	15,488	1,138	1,582,688
192,749	248,205	272,975	227,686	152,393	68,478	16,291	1,348	1,549,856
210,325	254,790	264,453	194,416	130,446	51,590	8,325	-	1,542,518
209,334	207,746	169,425	88,682	35,158	9,123	1,030	-	1,500,846
178,688	227,386	266,778	219,126	147,976	63,520	13,585	1,265	1,479,340
