# Supplementary material for: Nation-Wide Analysis of Glaucoma Medication Prescription in Fiscal Year of 2019 in Japan
Source: J Pers Med. 2022 Jun 11;12(6):956. doi: 10.3390/jpm12060956 (PMC9224924; doi:10.3390/jpm12060956)
Supplement: Supplementary file 1 [file jpm-12-00956-s001.zip › Supplementary files/File S2.pdf]

Age	Single	FDC	Brand	Generic	Man	Woman	Total	PG	β+CAI
0〜4	7,055	4,305	11,360	-	7,900	3,460	11,360	-	4,305
5〜9	26,971	10,904	37,875	-	21,908	15,967	37,875	4,671	10,904
10〜14	48,445	17,375	64,700	1,120	37,027	28,793	65,820	9,594	17,375
15〜19	71,851	32,651	95,124	9,378	53,629	50,873	104,502	14,933	30,043
20〜24	106,169	50,543	133,653	23,059	86,801	69,911	156,712	21,702	42,725
25〜29	186,150	88,054	226,004	48,199	152,449	121,755	274,203	48,672	67,302
30〜34	354,110	164,380	424,273	94,217	285,018	233,471	518,490	103,281	123,433
35〜39	699,746	306,845	827,921	178,670	556,867	449,724	1,006,591	213,982	220,843
40〜44	1,480,278	660,930	1,758,066	383,142	1,136,716	1,004,492	2,141,208	472,437	468,401
45〜49	2,671,652	1,215,341	3,209,652	677,340	2,032,389	1,854,603	3,886,992	858,225	846,335
50〜54	3,975,103	1,823,616	4,785,294	1,013,425	2,959,936	2,838,783	5,798,719	1,292,142	1,278,222
55〜59	5,482,682	2,537,594	6,616,075	1,404,201	4,142,750	3,877,526	8,020,276	1,802,329	1,797,506
60〜64	7,454,691	3,432,066	8,977,645	1,909,111	5,498,246	5,388,511	10,886,756	2,444,139	2,471,728
65〜69	11,631,434	5,253,065	14,039,579	2,844,920	8,265,620	8,618,879	16,884,499	3,808,058	3,812,352
70〜74	15,931,334	6,808,651	19,160,809	3,579,176	10,659,682	12,080,303	22,739,985	5,299,837	4,955,804
75〜79	19,758,168	7,950,426	23,843,119	3,865,475	12,374,600	15,333,994	27,708,594	6,566,429	5,761,791
80〜84	17,458,512	6,530,090	20,889,512	3,099,091	10,049,709	13,938,894	23,988,603	5,753,493	4,703,700
85〜89	12,837,832	4,387,615	15,140,769	2,084,678	6,322,241	10,903,206	17,225,447	4,205,155	3,154,759
90〜94	5,825,595	1,795,043	6,757,846	862,791	2,311,526	5,309,112	7,620,637	1,925,362	1,286,343
95〜99	1,246,676	329,908	1,399,693	176,891	359,853	1,216,731	1,576,584	426,478	231,704
100〜	110,132	19,018	118,256	10,894	16,154	112,996	129,150	39,298	13,300

α2	CAI	PG+β	ROCK	β	EP2	Other
-	3,815	-	3,240	-	-	-
5,850	8,855	-	7,595	-	-	-
11,545	13,470	-	8,125	4,376	-	1,335
18,215	13,480	2,608	10,698	6,519	3,131	4,875
28,265	16,535	7,818	16,000	10,727	5,170	7,770
45,545	29,443	20,752	22,445	18,563	9,877	11,605
86,980	49,645	40,947	40,534	33,539	19,201	20,930
171,710	96,588	86,002	79,765	65,072	39,034	33,595
365,725	199,945	192,529	159,370	137,024	77,582	68,195
675,368	380,341	369,006	279,925	237,222	123,412	117,159
1,017,528	591,686	545,394	415,413	324,346	153,545	180,443
1,413,309	841,060	740,088	585,624	420,786	170,062	249,512
1,939,325	1,219,138	960,338	795,718	546,790	179,334	330,247
3,009,132	1,996,561	1,440,713	1,251,434	805,924	209,334	550,991
4,060,407	2,804,746	1,852,847	1,670,308	1,093,944	207,746	794,346
5,144,350	3,477,617	2,188,635	2,095,228	1,255,221	169,425	1,049,898
4,631,925	3,141,004	1,826,390	1,824,983	1,001,598	88,682	1,016,827
3,424,539	2,399,844	1,232,856	1,328,448	645,559	35,158	799,129
1,509,156	1,140,613	508,700	567,498	272,266	9,123	401,577
292,407	261,865	98,204	103,245	62,996	1,030	98,655
20,560	26,011	5,718	8,190	4,868	-	11,205
